# Hepatic Dysfunction in Typhoid Fever During Pregnancy

**DOI:** 10.1155/IDOG/2006/64828

**Published:** 2006-06-12

**Authors:** Jorge Hasbun H., Raúl Osorio, Andrea Hasbun

**Affiliations:** ^1^Department of Obstetrics and Gynecology, Clinical Hospital, University of Chile, Avda Independence 1027, Santiago, Chile; ^2^Microbiology Programme, Biomedical Science Institute, Faculty of Medicine, University of Chile, Avda Independence 1027, Santiago, Chile

## Abstract

We described the hepatic dysfunction found in 10 cases out of 32 women with typhoid fever during pregnancy. This was associated with late diagnosis and maternal and perinatal complications.

## HEPATIC DYSFUNCTION IN TYPHOID FEVER DURING PREGNANCY

Chile has one of the best public health systems in Latin America, however the epidemiological control has not been successful in the eradication of typhoid fever (TF), this is due to the high prevalence of chronic carriers [1] and continuance to be an endemic area with the highest prevalence between 11 and 35 years of age, therefore the higher risk for pregnant women [2].

In 32 pregnant women with TF treated at University of Chile Clinical Hospital, between 1978–1998, we found 10 cases with jaundice, increased bilirubin: average 2.31 mg/dL, range 1.5–3.7, SD 0.69; increased aspartate aminotransferase (AST): average 118 IU, range 38–270, SD 67,4; increased alanine aminotransferase (ALT): average 123 IU, range 11–330, SD 109.74. Seven cases had liver enlargement.

After the treatment, eight patients were normal in both clinical and blood tests and continued a successful pregnancy. One case developed a cholestatic disease and a premature labour two weeks after the recovery of TF. Another case (no 7) presented a gastrointestinal bleeding during the fourth week (day 24) of bacteremia just at the beginning of therapy and then an adult respiratory distress syndrome and premature labor and delivery with neonatal death. She was treated in intensive care unit and had a prolonged hospital stay.

All the cases developed the hepatic dysfunction between days 8 and 24 of bacteremia, before the beginning of treatment. This suggests that the hepatic dysfunction is a late complication in illness evolution (Table 1).

The liver compromise of TF in adult patients has been described [3, 4], but there is no reference about this in TF during pregnancy [5, 6]. This was observed in 31.2% of the study cases (10/32) and in two cases associated with maternal and perinatal severe complications.

The prolonged exposure to toxins in the delayed diagnosis promotes the liberation of proinflammatory cytokines [7, 8], liver injury, and damaging host immunity [9]. So, it perpetuates the circulation of septic byproducts amplifyng tissue damage to others organs [10].

The finding of hepatic dysfunction in TF during pregnancy must be interpreted as a severe damage of cell function with potential progress to maternal multisystemic failure and perinatal death.

## Figures and Tables

**Table 1:** Hepatic dysfunction in typhoid fever at pregnancy. GA denotes gestational age (weeks), Hep (+) denotes liver enlargement, Bili denotes bilirrubin (mg/dL), AST denotes aspartate aminotransferase (IU), ALT denotes alanine aminotransferase (IU), and delay denotes days between the beginning of symptoms and therapy start.

Case no	GA	Hep	Bili	AST	ALT	Delay

1	30	+	1.95	64	64	11
2	33	−	1.68	180	155	9
3	32	+	3.76	270	200	12
4	23	+	2.8	117	330	18
5	31	+	3.15	85	11	22
6	32	+	1.84	140	63	10
7	31	+	1.98	83	111	24
8	28	+	2.0	57	27	12
9	18	−	1.9	182	295	9
10	33	−	2.8	84	87	8

## References

[1] Medina E, Yrarrazaval M (1983). Fiebre Tifoidea en Chile: Consideraciones Epidemiológicas [Typhoid fever in Chile: epidemiological considerations]. *Revista médica de Chile*.

[2] Hasbun J, Hasbun A (1999). Fiebre tifoidea y embarazo. *Revista chilena de obstetricia y ginecología*.

[3] Ayhan A, Gokoz A, Karacadag S, Telatar H (1973). The liver in typhoid fever. *The American Journal of Gastroenterology*.

[4] Khosla SN, Singh R, Singh GP, Trehan VK (1988). The spectrum of hepatic injury in enteric fever. *The American Journal of Gastroenterology*.

[5] Riggall F, Salkind G, Spellacy W (1974). Typhoid fever complicating pregnancy. *Obstetrics & Gynecology*.

[6] Seoud M, Saade G, Uwaydah M, Azoury R (1988). Typhoid fever in pregnancy. *Obstetrics & Gynecology*.

[7] Sartor RB (1994). Cytokines in intestinal inflammation: pathophysiological and clinical considerations. *Gastroenterology*.

[8] Wyant TL, Tanner MK, Sztein MB (1999). *Salmonella typhi* flagella are potent inducers of proinflammatory cytokine secretion by human monocytes. *Infection and Immunity*.

[9] Matuschak GM, Rinaldo JE (1988). Organ interactions in the adult respiratory distress syndrome during sepsis. Role of the liver in host defense. *Chest*.

[10] Rangel-Frausto MS, Pittet D, Costigan M, Hwang T, Davis CS, Wenzel RP (1995). The natural history of the systemic inflammatory response syndrome (SIRS). A prospective study. *JAMA: The Journal of the American Medical Association*.

